# Hemodynamic Collapse Following Therapeutic Plasma Exchange in a Patient Receiving an Angiotensin Receptor Blocker

**DOI:** 10.7759/cureus.7028

**Published:** 2020-02-18

**Authors:** Prashanth Ashok Kumar, Shweta Paulraj, Adaora Udekwu

**Affiliations:** 1 Internal Medicine, Upstate Medical University, Syracuse, USA; 2 Medicine, Upstate Medical University, Syracuse, USA

**Keywords:** therapeutic plasma exchange, albumin, angiotensin receptor blockers, ace inhibitors, drug-related side effects and adverse reactions, hypotension

## Abstract

Therapeutic plasma exchange (TPE) is a procedure for removal of plasma and its components while leaving behind cellular elements via an apheresis device. It is used in multiple conditions one among which is systemic lupus erythematosus (SLE). Adverse reactions from TPE range from mild hypotension and fever to life-threatening cardiovascular compromise. We report the case of sudden hemodynamic collapse following TPE for a neuropsychiatric lupus flare in a patient on losartan. A 62-year-old Caucasian female with a history of drug-induced lupus presented to the hospital with symptoms of a neuropsychiatric lupus flare. She was initiated on TPE with 5% albumin based on recommendations by her rheumatologist. Shortly after TPE, she became hypotensive with poor response to fluid boluses, requiring pressor support and intubation. These symptoms resolved within 24 hours on supportive measures. This was believed to be due to losartan use on the day of TPE. The medication was discontinued and she had further sessions of TPE with no complications. Angiotensin-converting enzyme (ACE) inhibitors have previously been associated with flushing and hypotension in patients undergoing TPE. Patients undergoing TPE have an activation of the prekallikrein and bradykinin system on contact with the extracorporeal membranes. ACE inhibitors potentiate this reaction by inhibiting bradykinin catabolism. Angiotensin receptor blockers (ARBs) have also been postulated to cause elevated bradykinin levels although data pertaining to the use of ARBs in TPE is limited. We hope to highlight this rare interaction in our case and emphasize the need for further data with regard to the same.

## Introduction

Therapeutic plasma exchange (TPE) is a procedure where plasma and its components are removed from the patient with the cellular components left untouched as blood passes through an apheresis device [1]. It was first employed in 1952 to treat hyper-viscosity in multiple myeloma [2]. TPE is ideal for removal of toxic substances such as antibodies, toxins or abnormal proteins. Conditions such as Guillain-Barre syndrome, myasthenia gravis, monoclonal gammopathies, thrombotic thrombocytopenic purpura (TTP), hemolytic uremic syndrome, idiopathic thrombocytopenia, and rheumatologic diseases like systemic lupus erythematosus (SLE) have seen the use of TPE [3]. One volume exchange roughly removes plasma equivalent to 65% of the intravascular space. The plasma removed is replaced with either albumin or fresh frozen plasma (FFP) [4].

Adverse reactions from TPE can range from the more common reactions such as mild hypotension, fever, chills to rare life-threatening complications such as cardiovascular and respiratory compromise. The estimated overall incidence is around 9% with a range of 1.5%-25% [3,5]. Reports have shown that patients taking angiotensin-converting enzyme (ACE) inhibitors who undergo TPE, especially with albumin replacement are at an increased risk of serious reactions like sudden hypotensive episodes [6].

We report a rare case of sudden hemodynamic collapse in a patient on losartan undergoing TPE with albumin for a neuropsychiatric lupus flare.

## Case presentation

A 62-year-old Caucasian female with a history of recurrent and resistant SLE, Crohn’s disease, hypertension, hyperlipidemia, coronary artery disease, and stroke was hospitalized for a neuropsychiatric flare of lupus. She presented with manic features like tangential, pressured speech, and agitation with difficulty in redirection. She was hemodynamically stable, not requiring oxygen support, alert and oriented on admission. Basic laboratory tests including blood counts, complete metabolic panel, and infectious work up including urinalysis and chest X-ray (CXR) were negative.

Ten years prior, she developed drug-induced lupus after the use of infliximab for Crohn’s disease which resolved on discontinuing the medication. Her disease was stable up until two years ago, since when she has had recurrent episodes of lupus-associated serositis causing severe cardiorespiratory symptoms. She continued to have recurrent exacerbations despite therapy with steroids, hydroxychloroquine, mycophenolate, belimumab, and cyclosporine. Two weeks prior to her presentation, she developed aphasia, seizures, and mania which improved with steroids and plasma exchange. She was started on antiepileptics, mood stabilizers, and antipsychotics. She underwent extensive rheumatologic and neurologic work up including magnetic resonance imaging (MRI) of the brain, lumbar puncture, and electroencephalography (EEG) which were unremarkable. She was evaluated by her rheumatologist on the day of admission and had been recommended initiation of plasmapheresis as she had failed multiple treatment regimens in the past.

Her home medications included amlodipine and losartan for hypertension among several others. All her home medications were resumed on admission. She had received 25 mg of losartan along with her usual morning medications. A few hours later, she was started on single volume TPE with 5% albumin as replacement fluid. Shortly after TPE, she decompensated becoming severely hypotensive. Her blood pressure did not respond to fluid resuscitation and she required both epinephrine and norepinephrine support. She had to be intubated for respiratory compromise. Blood gases revealed hypoxia and labs were unremarkable except for hypocalcemia. With supportive measures, she improved in 24 hours, was extubated and no longer required vasopressor support. Losartan was discontinued following the episode. Through her hospital course, she received four more sessions of TPE with 5% albumin and tolerated it well without any complications. She was also treated with rituximab resulting in improvement of her neuropsychiatric symptoms.

## Discussion

Our case is a rare presentation of severe hemodynamic decompensation following TPE with albumin. Severe reactions following plasma exchange is an uncommon occurrence [5,6].

Owen et al., in their study, analyzed 299 patients undergoing TPE and found that 4.7% received ACE inhibitors and all of them had reactions characterized by flushing and hypotension. They also mention that patients receiving ACE inhibitors and undergoing TPE with albumin have a 100% risk of having such a reaction [6]. The vasodilatory and blood pressure lowering effect of bradykinin is already well established [7]. It is also equally well known that ACE inhibitors are associated with elevated levels of bradykinin [8]. Perseghin et al. studied the bradykinin levels in volunteers following donor plasmapheresis and found the levels to be higher in those who were receiving an ACE Inhibitor. They observed that patients on ACE inhibitors showed greater variations in systolic blood pressure than those who were not on it [9]. It is hypothesized that blood comes in contact with extracorporeal membranes in procedures like TPE that carry a negative or sometimes, a positive charge. Charged materials through Factor 12, also called Hageman factor may cause activation of the prekallikrein and bradykinin system. It is postulated that this interaction may be responsible for life-threatening reactions like cardiovascular compromise. ACE inhibitors having a synergistic action on the bradykinin pathway may increase the likelihood of such reactions. Thus, a plausible hypothesis for such a catastrophic reaction as seen in our patient may be due to elevated levels of bradykinin [9].

Studies have shown that hypotensive reactions are more common when albumin is used as the replacement fluid. The choice of whether to use 5% albumin or FFP is disease specific. FFP is used in coagulopathies like TTE and hemolytic uremic syndrome where clotting factors have clinical benefits. Albumin is used in all other conditions due to the lower possibility of allergic reactions and antibody development. Albumin preparations were found to contain variable amounts of kallikrein activating substances. Rapid infusion of albumin containing kallikrein activators may lead to sudden surges in bradykinin levels. This in combination with drugs that inhibit bradykinin catabolism, brews the perfect recipe for causing hemodynamic compromise. Strauss et al. recommend stopping ACE inhibitors 24-48 hours prior to TPE depending on the half-life of the medication; whereas, Owen et al. recommend a period of 24 hours [2,6]. Longer acting drugs may need a period of up to 72 hrs [10].

While ARBs, in the ideal sense, do not cause bradykinin elevation, literature shows that ARBs like losartan cause elevated bradykinin levels. By blocking the receptor, ARBs cause a reactive increase in angiotensin II, ultimately leading to increased bradykinin levels. It is also not uncommon to see patients with ACE inhibitor-induced angioedema develop similar reactions to ARBs [11-13]. Data with regard to ARBs and TPE is lacking with no case reports or trials and thus entails further research.

## Conclusions

ARBs and ACE inhibitors are one of the most commonly used classes of drugs for hypertension. There is no policy with regard to patients on ARBs and ACE inhibitors undergoing plasma exchange in most hospitals. On most occasions, hospitalists and primary care physicians manage a patient’s medications. Given the lack of awareness on this interaction, there is an increased risk of adverse events, as seen in our case, in patients needing TPE. Through our case, we hope to increase the awareness regarding this rare interaction and stress on the need for more data and research, especially on ARBs and plasma exchange.
